# Chromosome Asynapsis Is the Main Cause of Male Sterility in the Interspecies Hybrids of East Asian Voles (*Alexandromys*, Rodentia, Arvicolinae)

**DOI:** 10.3390/genes14051022

**Published:** 2023-04-30

**Authors:** Tatiana Bikchurina, Marina Pavlenko, Elena Kizilova, Daria Rubtsova, Irina Sheremetyeva, Irina Kartavtseva, Anna Torgasheva, Pavel Borodin

**Affiliations:** 1 Department of Cytology and Genetics, Faculty of Natural Sciences, Novosibirsk State University, 630090 Novosibirsk, Russia; 2 Institute of Cytology and Genetics, Siberian Branch of the Russian Academy of Sciences, 630090 Novosibirsk, Russia; 3 Federal Scientific Center of the East Asia Terrestrial Biodiversity, Far East Branch of the Russian Academy of Sciences, 690022 Vladivostok, Russia

**Keywords:** hybrid sterility, gray voles, chromosomal polymorphism, synaptonemal complex, crossing over

## Abstract

Closely related mammalian species often have differences in chromosome number and morphology, but there is still a debate about how these differences relate to reproductive isolation. To study the role of chromosome rearrangements in speciation, we used the gray voles in the *Alexandromys* genus as a model. These voles have a high level of chromosome polymorphism and substantial karyotypic divergence. We investigated testis histology and meiotic chromosome behavior in the captive-bred colonies of *Alexandromys maximowiczii*, *Alexandromys mujanensis*, two chromosome races of *Alexandromys evoronensis*, and their interracial and interspecies hybrids, to explore the relationship between karyotypic differences and male hybrid sterility. We found that the seminiferous tubules of the males of the parental species and the interracial hybrids, which were simple heterozygotes for one or more chromosome rearrangements, contained germ cells at all stages of spermatogenesis, indicating their potential fertility. Their meiotic cells displayed orderly chromosome synapsis and recombination. In contrast, all interspecies male hybrids, which were complex heterozygotes for a series of chromosome rearrangements, showed signs of complete sterility. Their spermatogenesis was mainly arrested at the zygotene- or pachytene-like stages due to the formation of complex multivalent chains, which caused extended chromosome asynapsis. The asynapsis led to the silencing of unsynapsed chromatin. We suggest that chromosome asynapsis is the main cause of meiotic arrest and male sterility in the interspecies hybrids of East Asian voles.

## 1. Introduction

Closely related species of mammals often differ in chromosome number and morphology [1]. It is still under discussion whether these karyotypic differences are the cause or consequence of speciation [2]. The classical version of the chromosome speciation hypothesis proposes that the hybrid sterility of the heterozygotes for chromosome rearrangements results from impaired synapsis, recombination, and segregation of the homeologous chromosomes [3]. A modern version emphasizes the suppression of recombination around the breakpoints of the rearrangements in the hybrids, which could reduce gene flow across hybrid zones, promote divergence of parental populations, and, ultimately, lead to speciation [4,5,6].

The severity of the meiotic aberrations depends on the degree of chromosomal heterozygosity and the complexity of the expected synaptic configurations. Simple heterozygosity for single inversion or chromosome fusion is unlikely to cause meiotic disturbances and sterility. Even multiple simple heterozygosity, when several heteromorphic bivalents or trivalents are present in the same meiotic cell, do not usually lead to disruption in chromosome synapsis and segregation [7,8]. On the contrary, complex heterozygotes for several fusions that share common chromosome arms and/or several inversions in the same chromosome often exhibit serious meiotic abnormalities [2,8,9,10,11,12,13,14,15]. Analyzing the sequential steps of karyotypic evolution from individual variation to the divergence between populations, chromosomal races, and, finally, species is particularly important for understanding the role of chromosome rearrangements in speciation.

Eastern gray voles of the genus *Alexandromys*, including *A. maximowiczii* Ognev, 1914, *A. mujanensis* Orlov, and Kovalskaja, 1978, and two chromosome races of *A. evoronensis*, Kovalskaya and Sokolov, 1980, provide a good model for this study. They all exhibit an exceptionally high level of chromosomal polymorphism, population polytypism, and advanced karyotypic divergence from each other.

*A. maximowiczii* (MAX) has a wide but mosaic distribution [16,17]. It demonstrates both intra- and interpopulation polymorphism for a series of centromeric shifts, inversions, fusions, and whole-arm translocations. Its diploid chromosome number (2n) varies from 36 to 44. The number of chromosome arms (FN) varies from 52 to 62 [18,19].

*A. mujanensis* (MUJ) is an endemic species of the northern regions of Buryatia and the northwest of the Trans-Baikal Territory [20]. It is polymorphic for pericentric inversions or centromeric shifts in several chromosome pairs (2n = 38; FN = 50–53) [21,22,23,24].

*A. evoronensis* has been found in several scattered localities in the Khabarovsk and Amur provinces [25,26]. It is conditionally subdivided into two chromosomal races: “Evoron” (EVE) (2n = 38–41; FN = 54–59) inhabiting the Evoron-Chukchagir lowland, and “Argi” (EVA) (2n = 34, 36, 37; FN = 51–56) distributed along the Argi River, the Zeya River tributary, and along the Urgal River, the tributary of the Upper Bureya River [27,28].

The time of divergence between these three species is estimated to have been approximately 110 thousand years ago [29,30], yet their interspecies hybrids, of both sexes, have been shown to be sterile [21,31].

The aim of this study was to identify the cytological mechanisms of male hybrid sterility using the specimens from the laboratory colonies of MAX, MUJ, EVE, and EVA, and their F1 hybrids. To estimate the reproductive potential of the purebred, interracial, and interspecies hybrid males and reveal the stage of spermatogenic arrest, we performed a conventional histological analysis of their testes. We also used TdT-mediated dUTP nick-end labeling (TUNEL) to detect apoptotic germ cells. To assess correspondence between spermatogenesis dynamics and meiotic chromosome behavior, we immunolocalized SYCP3 (protein of lateral elements of synaptonemal complexes, SC), SYCP1 (protein of the central element of SC), centromere proteins, and MLH1 (mismatch repair protein that marks mature recombination nodules) at the surface spreads isolated from the spermatocytes of adult males [32]. Unrepaired DNA double-strand breaks (DSBs) at the zygotene and early pachytene stages and chromosome regions where meiotic silencing of unsynapsed chromatin (MSUC) occurs were identified through immunolabeling of phosphorylated H2A.X histone (γH2A.X) [33,34].

## 2. Materials and Methods

### 2.1. Specimens

The founders of the captive-bred colonies were captured using live traps at the locations listed in Table S1. The colonies of MAX MUJ, EVA, and EVE were established and maintained by outcrossing within each colony in the animal house of the Federal Scientific Center of East Asia Terrestrial Biodiversity (Vladivostok), under natural daylight and conventional conditions. The interracial and interspecies hybrids were produced from the crosses listed in Table S1 and were also maintained under these conditions. A total of 13 males of the parental species, 2 interpopulation, 3 interracial, and 11 interspecies hybrids were examined in this study. 

The animals were handled, maintained, and euthanized in accordance with the approved national guidelines for the care and use of laboratory animals. All experiments were approved by the Ethics Committee on Animal Care and Use of the Institute of Cytology and Genetics (approval No. 132 of 19 October 2022) and the Committee for the Regulation of Experimental Research of the Federal Scientific Center of the East Asia Terrestrial Biodiversity (approval No. 1 of 25 April 2022).

### 2.2. Histological Analysis

The left testes of adult males were isolated immediately after euthanasia, cut into 5–7 mm^3^ pieces, and fixed in 10% buffered formalin for 2–3 weeks. The samples were dehydrated in a graded ethanol series, immersed in xylene, and embedded in paraffin. Then, 6-μm-thick sections were cut using a rotary microtome equipped with a Section Transfer System (Microm HM 355 S, Thermo Fisher Scientific Microm International GmbH, Walldorf, Germany) and mounted on slides. The sections were deparaffinized, stained with hematoxylin and eosin, and examined under an Axioskop 2 Plus microscope (Carl Zeiss, Jena, Germany) equipped with a CCD camera AxioCam HRc (Carl Zeiss) and AxioVision image-processing package (Carl Zeiss). The stages of seminiferous epithelium at the cross sections of the testes were identified according to Russell et al. [35] and Ahmed and de Rooij [36]. According to Russell et al. [35] the stages VI-VIII of the seminiferous epithelium cycle in the classical model species (mouse and rat) have the same pattern of cell stages: elongated spermatids of the last stages, round spermatids, pachytene spermatocytes, and spermatogonia. Galleni et al. [37] showed that the general morphology of the gonads of *Microtus savii* and *Microtus brachycercus* was the same as in mice and rats. Thus, restricting the tubule morphometry to only stages VI-VIII lets us minimize the taxon-specific variation in terms of stages. Different stages of prophase I (leptotene, zygotene, pachytene, and diplotene) were distinguished by the state of chromatin in the nuclei and the stage of the tubule.

The spermatid-to-spermatocyte I ratio was assessed in randomly chosen tubules in stages VI-VIII. A significant deviation of the observed from the expected 4:1 ratio was considered an indicator of spermatogenic abnormalities.

### 2.3. Detection of Apoptotic Cells in Seminiferous Tubules

The deparaffinized sections were washed in PBS and fixed in 4% paraformaldehyde. Apoptosis was analyzed using the DeadEnd Fluorometric TUNEL System (Promega, Madison, WI, USA) according to the manufacturer’s protocol. Slides were mounted with Vectashield Antifade mounting medium (Vector Laboratories, Burlingame, CA, USA) to reduce fluorescence fading and examined under the Axioscop 2 Plus microscope as described above. The apoptotic index was calculated as the ratio of the number of TUNEL-positive spermatogonia and spermatocytes to the total number of spermatogonia and spermatocytes observed in 25 randomly-chosen tubules per specimen.

### 2.4. SC Spreading for Light and Electron Microscopy

Chromosome spreads for SC analysis were prepared by the drying-down technique [38] from the right testes of adult males. The number of males examined is shown in Table S2. Immunostaining was performed according to the protocol described by Anderson et al. [32] using rabbit polyclonal anti-SYCP3 (1:500; Abcam, cat. No. ab15093), mouse monoclonal anti-SYCP3 (1:80; Abcam, Cambridge, UK, cat. No. ab97672), mouse monoclonal anti-MLH1 (1:70; Abcam, cat. No. ab14206), rabbit polyclonal anti-MLH1 (1:70; Abcam, cat. No. ab92312), rabbit polyclonal anti-SYCP1 (1:80; Abcam, cat. No. ab15090), rabbit polyclonal anti-γH2A.X (1:500; Abcam, cat. No. ab2893) primary antibodies and human anti-centromere (ACA) (1:100; Antibodies Inc, Davis, CA, USA, cat. No. 15–234) primary antibodies. The secondary antibodies used were Cy3-conjugated goat anti-rabbit (1:500; Jackson ImmunoResearch, Philadelphia, PA, USA, cat. No. 111-165-144), Cy3-conjugated donkey anti-mouse (1:170; Jackson ImmunoResearch, cat. No. 715-165-151), FITC-conjugated goat anti-mouse (1:50; Jackson ImmunoResearch, cat. No. 115-095-003), FITC-conjugated donkey anti-rabbit (1:30; Jackson ImmunoResearch, cat. No. 711-095-152), and AMCA-conjugated donkey anti-human (1:70; Jackson ImmunoResearch, cat. No. 709-155-149). Antibodies were diluted in PBT (3% bovine serum albumin and 0.05% Tween 20 in phosphate-buffered saline). A solution of 10% PBT was used for blocking. Primary antibody incubations were performed overnight in a humid chamber at 37 °C; and secondary antibody incubations, for 1 h at 37 °C. Slides were mounted in a Vectashield antifade mounting medium (Vector Laboratories, cat. No. H-1000-10).

The preparations were visualized with an Axioplan 2 imaging microscope equipped with a CCD camera (CV M300, JAI Corporation, Yokohama, Japan), CHROMA filter sets, and the ISIS4 image-processing package (MetaSystems GmbH, Altussheim, Germany). The brightness and contrast of all images were enhanced using Corel PaintShop Pro X6 (Corel Corp, Ottawa, ON, Canada).

The centromeres were identified by ACA foci. MLH1 signals were only scored if they were localized on SCs. The number of MLH1 foci was scored in the mid-late pachytene cells. In the case of purebred specimens and intraspecies hybrids, we considered pachytene as the stage when more than 90% of autosomal bivalents were synapsed. In the case of the interspecies hybrids, we considered the stage as “pachytene-like”, when most parts of chromosomes were synapsed. The number and morphology of the SC elements were used to estimate the 2n and FN of the specimens.

For electron microscopic examination, the SC spreads were stained with silver nitrate [39] and covered with a plastic film. After light microscopic examination, the spreads were transferred to specimen grids and examined under an electron microscope JEM-1400 (JEOL, Tokyo, Japan) at 80 kV.

### 2.5. Data Analysis

The data in this paper are presented as mean values and standard deviations (mean ± SD). A pairwise Wilcoxon test was used to compare the observed and expected spermatid-to-spermatocyte ratios and apoptotic index. Welch’s two-sample *t*-test was used to compare the total number of MLH1 signals per cell. All analyses were carried out in the R (v4.1.3) environment [40].

## 3. Results

### 3.1. Histological Analysis

To evaluate the reproductive potential of the males under study, the degree of disturbances in spermatogenesis, and the level of germ-cell apoptosis, we used a conventional histological analysis of the testes and the TUNEL test.

#### 3.1.1. Parental Species, Interpopulation, and Interracial Hybrids

**Figure 1:**
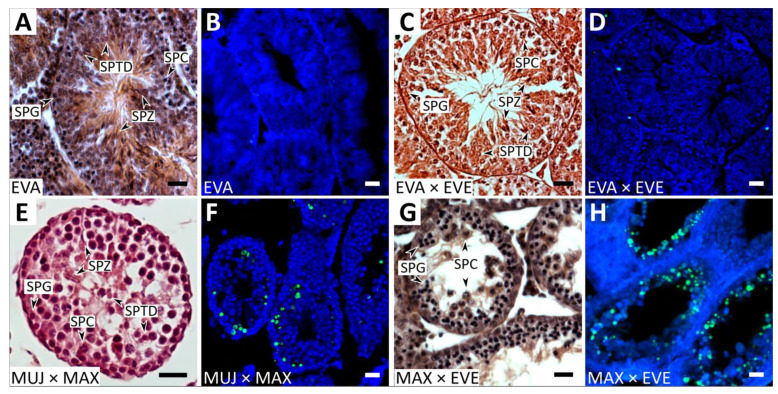
Histological sections of testes of adult purebred *A. evoronensis* “Argi”, EVA (**A**,**B**), interracial hybrid *A. evoronensis* “Argi” × *A. evoronensis* “Evoron”, EVA × EVE (**C**,**D**), interspecies hybrids *A. mujanensis* × *A. maximowiczii*, MUJ × MAX (**E**,**F**), and *A. maximowiczii* × *A. evoronensis* “Evoron”, MAX × EVE (**G**,**H**) after staining by hematoxylin–eosin (**A**,**C**,**E**,**G**) and detection of apoptotic cells using TUNEL (green) and DAPI counterstaining (blue) (**B**,**D**,**F**,**H**). SPG, spermatogonium; SPC, spermatocyte; SPTD, spermatid; SPZ, spermatozoon. Bar: 20 μm.

We detected no abnormalities in the seminiferous epithelium cell cycle of all purebred males and interpopulation hybrids EVA1 × EVA2 (Figure 1A,B). First, *A. evoronensis* “Argi” (EVA1) stock was derived from the specimens trapped near Chegdomyn village whereas the EVA2 specimen was derived from specimens trapped near the Argi River’s banks (Table S1). The mean spermatid-to-spermatocyte I ratio in EVA1 × EVA2 was 3.61:1, which did not differ significantly from that observed in the purebred EVA males (3.35:1) or from the expected 4:1 ratio (Tables S2 and S3; pairwise Wilcoxon test, *p* = 0.44). The TUNEL assay revealed a small number of apoptotic cells in some tubules of these males (Tables S2 and S4), while in most tubules no signal was detected (Figure 1B).

In two out of three interracial EVA × EVE hybrids examined, we observed the same pattern (Figure 1C,D). The apoptotic index in these interracial hybrids was 0.005. It did not differ from that in the parental specimens (0.002 of parental species, pairwise Wilcoxon test, *p* = 0.086). However, in one EVA × EVE hybrid, spermatogenesis advanced to the early spermatocyte stage only. Its spermatid-to-spermatocyte ratio was significantly lower than that of its siblings (0.05:1), pairwise Wilcoxon test, *p* = 8.4 × 10^−12^).

#### 3.1.2. Interspecies Hybrids

All interspecies hybrids showed varying degrees of disturbances in spermatogenesis. Germ cells in different seminiferous tubules of the MUJ × MAX hybrid progressed to different stages of spermatogenesis, from spermatocytes to elongated spermatids, although in low numbers (Figure 1E; Tables S2 and S3). Round spermatids were solitary or aggregated into multinuclear cell elements of various sizes, similar to those described by Meyer et al. [21] in MUJ × MAX males. The spermatid-to-spermatocyte I ratio was significantly reduced in the MUJ × MAX hybrids (1.35:1) compared to that observed in the purebred males (pairwise Wilcoxon test, *p* = 4.7 × 10^−8^). This indicates that spermatogenesis was partially halted before spermatid formation. The apoptotic index in the hybrid males (0.037) was significantly higher than that of the purebred males of the parental species (0.002) (pairwise Wilcoxon test, *p* = 4.3 × 10^−8^).

The testis sections of one EVA × MUJ hybrid were rather similar to those of the MUJ × MAX. The difference concerned the number and appearance of spermatids. They were less numerous and formed fewer multinuclear cell elements. We detected a few immature spermatozoa. In another EVA × MUJ hybrid, spermatogenesis was arrested at the pachytene stage. We observed the same pattern in the reciprocal hybrid male MUJ × EVA. The mean spermatid-to-spermatocyte I ratio in the hybrids resulting from both crosses was significantly lower (0.15:1) than in the purebred males, whereas the apoptotic index (0.033) was significantly higher (pairwise Wilcoxon test, *p* = 7.9 × 10^−14^ and 5.1 × 10^−15^, respectively).

In MAX × EVE hybrids, spermatogenesis progressed until the pachytene in most of the tubules examined. It was accompanied by apoptosis (Figure 1G,H). In two of four EVA × MAX hybrids, spermatogenesis was stopped at the spermatocyte I stage, the number of spermatocytes was reduced, and no spermatids were observed. In two other hybrids, spermatogenesis advanced to the spermatid stage. We observed a few spermatids, isolated or clamped into multinuclear cell elements. No sperm were detected.

The mean spermatid-to-spermatocyte I ratios in the MAX × EVE hybrids (0.05:1) and in the EVA × MAX hybrids (0.13:1) were significantly lower in comparison with the purebred males of the parental species (pairwise Wilcoxon test, *p* = 2.0 × 10^−15^ and *p* < 10^−16^, respectively). The apoptotic index for the EVA × MAX cross was significantly elevated compared to the parental species (0.023 vs. 0.002, pairwise Wilcoxon test, *p* = 1.2 × 10^−12^). There was no difference in the apoptotic index between all interspecies hybrids examined (Tables S2 and S4; pairwise Wilcoxon test, *p* > 0.05).

Thus, conventional histological analysis and the TUNEL test results show that the testes of all purebred males, interpopulation, and all but one of the interracial hybrids contained germ cells at all stages of spermatogenesis, indicating the males’ potential fertility. However, all interspecies male hybrids demonstrated all signs of complete sterility. Their spermatogenesis was arrested mainly at the zygotene- or pachytene-like stage, followed by the apoptosis of the germ cells, and no mature sperm were detected.

### 3.2. Cytological Analysis

To gain insight into the cytological basis of the spermatogenic aberrations described above, we examined chromosome synapsis, recombination, and epigenetic modifications in the interpopulation, interracial, and interspecies hybrids in comparison to the parental species. The chromosome arms in the voles are difficult to distinguish using conventional karyotyping [19,23]. For this reason, we assessed types of rearrangements based on the synaptic configurations they form in the heterozygotes.

**Figure 2:**
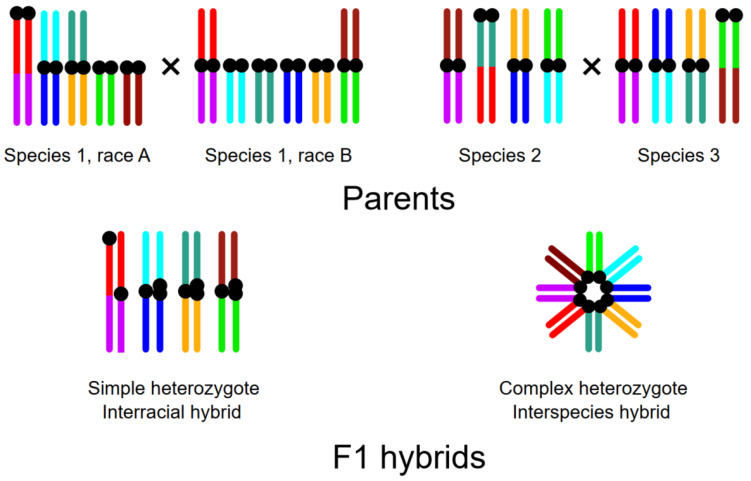
Expected synaptic configurations in the parental species or races and their F1 hybrids: simple and complex heterozygotes.

The specimens involved in the analysis differed in the type and complexity of the expected synaptic configurations (Figure 2). The purebred males of the parental species were either karyotypic homozygotes or simple heterozygotes for one or several rearrangements: chromosome fusions, translocations, pericentric inversions, and/or centromere shifts. In the pachytene cells of the karyotypically homozygous males, we expected to observe normally synapsed bivalents. The cells of the simple heterozygotes for chromosome fusions should display trivalents involving metacentric chromosomes and their twin acrocentric partners [11,12]. The simple heterozygotes for inversions or centromere shifts would contain heteromorphic bivalents with inversion loops or with misaligned centromeres [41,42,43]. All interspecies hybrids were complex heterozygotes for many chromosome rearrangements that shared common chromosome arms. Homologous synapsis of their chromosomes would result in a complex multivalent: chains or rings involving many chromosome arms [6,8,15].

#### 3.2.1. Parental Species

##### *Alexandromys maximowiczii* (MAX)

**Figure 3:**
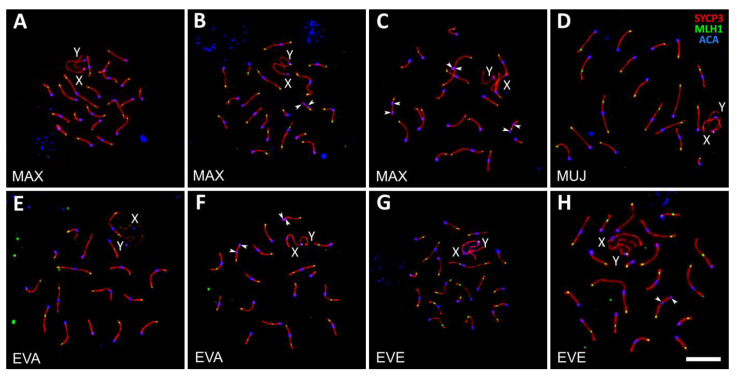
Pachytene spermatocytes of *A. maximowiczii*, MAX (**A**–**C**), *A. mujanensis*, MUJ (**D**), *A. evoronensis* “Argi”, EVA (**E**,**F**), and *A. evoronensis* “Evoron”, EVE (**G**,**H**) homozygous (**A**,**D**,**E**,**G**) and heterozygous (**B**,**C**,**F**,**H**) for chromosome rearrangements after immunolocalization of SYCP3 (red), MLH1 (green), and centromeric proteins (blue). Arrowheads show the centromeres of heteromorphic bivalents. Bar: 10 µm.

**Figure 4:**
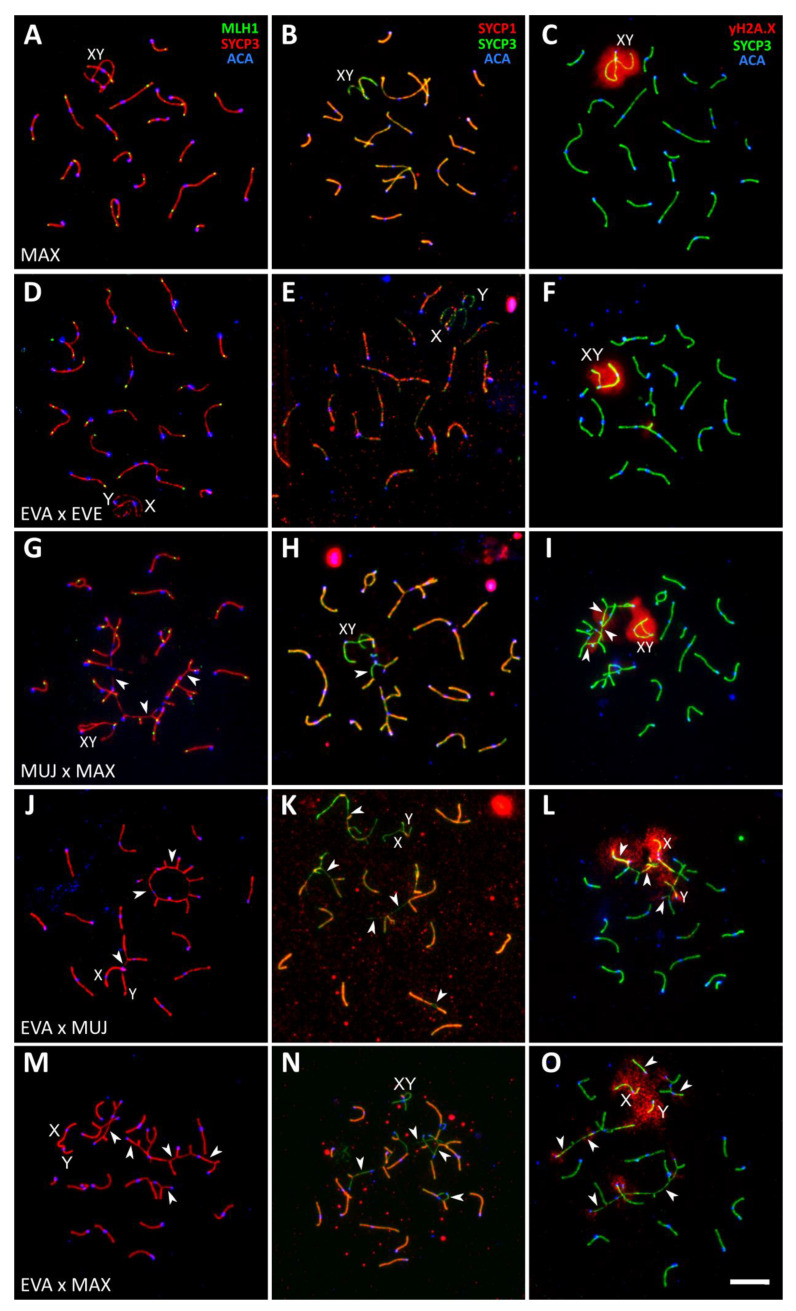
Pachytene and pachytene-like spermatocytes of *A. maximowiczii*, MAX (**A**–**C**), *A. evoronensis* “Argi” × *A. evoronensis* “Evoron”, EVA × EVE (**D**–**F**), *A. mujanensis* × *A. maximowiczii*, MUJ × MAX (**G**–**I**), *A. evoronensis* “Argi” × *A. mujanensis*, EVA × MUJ (**J**–**L**), and *A. evoronensis* “Argi” × *A. maximowiczii*, EVA × MAX (**M**–**O**) after immunolocalization of SYCP3, SYCP1, MLH1, γH2A.X, and centromeric proteins (ACA). Arrowheads show asynapsed SC regions of the autosomes. Bar: 10 µm.

Two out of four MAX males examined were homozygous for all chromosomes polymorphic in this species. In these males, we observed orderly synapsis and recombination of autosomes and sex chromosomes. Almost all autosomal bivalents were completely synapsed. A pair of univalents was detected at the mid-late pachytene stage in 1 out of 61 cells of one male (Figure 3A and Figure 4A–C). Most bivalents had at least one MLH1 focus (Figure 3A and Figure 4A). The X- and Y-chromosomes synapsed with each other, forming the sex body (Figure 3A and Figure 4A–C). It was heavily labeled by γH2A.X antibodies, indicating its silencing (Figure 4C).

Two other MAX males were heterozygous for chromosome variants. In the pachytene spreads of one of them, we observed completely paired heteromorphic bivalent of medium size containing two misaligned centromeres (Figure 3B). We did not observe MLH1 foci in the region between the centromeres, while another part of the bivalent always contained one MLH1 focus. Such a distribution of MLH1 foci, which mark crossover sites, occurs in heterozygotes for pericentric inversion when the inverted region is nonhomologously synapsed with a noninverted partner, and therefore crossing over is completely suppressed [43].

In the pachytene cells of the fourth MAX male, we detected three heteromorphic bivalents (Figure 3C). Two of them (one medium and another small) contained one centromere at the end of the bivalent and another centromere in the middle. This indicates heterozygosity for pericentric inversion or centromeric shift. The third heteromorphic bivalent was a large metacentric containing a hairpin in the pericentromeric region, with one centromere located at the top of the hairpin and another at its base. Probably it indicates a heterozygosity for the amplification of pericentric chromatin. The size of the hairpin varied between the cells. In some cells, it was absent, and the centromeres were aligned due to synaptic adjustment (Figure S1). Similar meiotic configurations have been detected in several males of *Microtus arvalis* [44].

The average number of MLH1 foci per cell in MAX males was 23.9 ± 2.2.

##### *Alexandromys mujanensis* (MUJ)

All three MUJ males analyzed in this experiment were homozygous for all polymorphic chromosomes. They showed orderly synapsis and recombination of all autosomes and sex chromosomes similar to those described above for MAX homozygotes (Figure 3D). The average number of MLH1 foci was 20.2 ± 1.6.

##### *Alexandromys evoronensis* “Argi” (EVA)

Two males of EVA2 derived from specimens trapped near the banks of the Argi River (Table S1) were homozygous for all polymorphic chromosomes in this race. All its 17 autosomal bivalents and the XY bivalent showed normal synapsis and recombination at the pachytene stage (Figure 3E). We did not detect autosomal univalents in any of the cells examined (Table S2).

Two males of EVA1 stock derived from the specimens trapped near Chegdomyn village were heterozygous for pericentric inversions or centromeric shifts in two autosomal pairs of medium size. One pair involved middle-sized metacentric and acrocentric chromosomes and another one consisted of a medium-sized submetacentric chromosome and an acrocentric one. No MLH1 foci were detected between the centromeres of the heteromorphic bivalents (Figure 3F). In both simple heterozygotes, the rearranged chromosomes were completely synapsed at the pachytene. We did not observe γH2A.X labeling of the heteromorphic synaptic configurations. This indicates that the rearranged homologs have completed synapsis and DSB repair, and their carriers can pass a checkpoint at the zygotene-pachytene transition.

Two interpopulation hybrid males were produced by crossing EVA2 derived from the voles trapped near the banks of the Argi River with EVA1 stock derived from the voles trapped near Chegdomyn village (Table S1). The distance between these localities is about 450 km as the crow flies. One of the hybrids had four; another had three heteromorphic bivalents, containing misaligned centromeres (Figure S2A). These bivalents indicate simple heterozygosity for centromere shifts, or pericentric inversions, or the chromosomes, which probably resulted from centromere–centromere fusions in one population and centromere–telomere fusions in another population. The average number of MLH1 foci per cell in all EVA males examined was 20.7 ± 2.0. as The interpopulation hybrids did not show a significant difference in comparison with the EVA specimen (Welch’s *t*-test, *p* = 0.17).

##### *Alexandromys evoronensis* “Evoron” (EVE)

One male was homozygous for all the polymorphic chromosomes in this race. All its 19 autosomal bivalents and the XY bivalent showed normal synapsis and recombination in mid-to-late pachytene cells (Figure 3G; Tables S2, S5 and S6). Another male was heterozygous for a centromeric shift in a medium-sized autosome (Figure 3H). All its pachytene cells contained a bivalent with one centromere at the end and another in the middle. In some cells, we observed single MLH1 foci between the centromeres. The average number of MLH1 foci in the EVE males was 22.0 ± 1.1.

Thus, the simple heterozygotes for the chromosomes, polymorphic in local populations, show complete chromosome synapsis and approximately the same level of meiotic recombination as the standard homozygotes. These observations, together with the results of histological analysis, indicate that their fertility should not be affected.

#### 3.2.2. Interracial Hybrids

We examined two interracial male hybrids between EVA2 stock (derived from the voles trapped near the banks of the Argi River) and EVE stock (derived from the voles trapped near Evoron Lake) (Table S1). The distance between these localities is about 500 km as the crow flies, which is approximately the same as between the two populations of EVA, described above. Yet, the chromosome heterozygosity of the interracial hybrids was higher. Two interracial hybrids (one with normal spermatogenesis and one with delayed spermatogenesis) showed exactly the same sets of heteromorphic synaptic configurations: two bivalents with misaligned centromeres and four trivalents with pronounced side arms (Figure 4D–F). Thus, the arrest of spermatogenesis in the latter male was probably due to some environmental factor rather than chromosomal incompatibility.

One heteromorphic bivalent probably resulted from a centromeric shift in one of the pairing partners, since we regularly observed recombination nodules between its misaligned centromeres (Figure S2B_a_). The centromeres of another heteromorphic bivalent were located rather close to each other to accommodate recombination nodules (Figure S2B_b_). For this reason, we cannot distinguish between the possibilities of centromeric shift and pericentric inversion.

Two trivalents occurred due to homologous synapsis between the twin acrocentric chromosomes with metacentric (Figure S2B_c_) or acrocentric (Figure S2B_d_) chromosomes, which probably resulted from the centromeric fusion of their homologs in one race and centromere–telomere fusion in another race. The origin of the two other trivalents is more complex. In one of them, the metacentric partner apparently resulted from the centromeric fusion of two acrocentrics followed by a pericentric inversion (Figure S2B_e_). Another complex trivalent resulted from the pairing of three metacentric chromosomes. The largest of them had probably occurred due to a fusion of two acrocentric chromosomes, both of which became metacentric due to the fixation of the pericentric inversions (Figure S2B_f_).

One more hybrid with normal spermatogenesis had the same four trivalents and one bivalent with grossly misaligned centromeres.

In the early and middle pachytene cells, we observed partial asynapsis in the pericentromeric regions of the trivalents. These regions were labeled with γH2A.X antibodies, indicating a transcriptional inactivation of unpaired chromatin. However, by the late pachytene, the proximal parts of the twin acrocentrics were usually completely paired, either homologously with the metacentric partner or nonhomologously with each other, forming side arms of various lengths. In the late pachytene cells, we did not detect γH2A.X signals at the trivalents. At this stage, γH2A.X signals were detected at the sex body only (Figure 4F).

The average number of late recombination nodules per cell labeled with MLH1 in the EVA × EVE hybrids was similar to that in one parental race, (EVA) Welch’s test, *p* = 0.94, and significantly lower than in other race (EVE) (Table S2; Welch’s test, *p* < 10^−13^).

Thus, a substantial chromosomal divergence between the local populations and chromosome races within *A. evoronensis* and the occurrence of a large number of heteromorphic synaptic configurations in their F1 hybrids (simple heterozygotes) do not lead to chromosome-pairing failure, a decrease in recombination frequency, and meiotic silencing of the unsynapsed chromatin.

#### 3.2.3. Interspecies Hybrids

Chromosome synapsis was severely disrupted in all interspecies hybrids. However, the severity of the disruption varied between the crosses, between the individuals within the crosses, and between the germ cells within the individuals.

**Figure 5:**
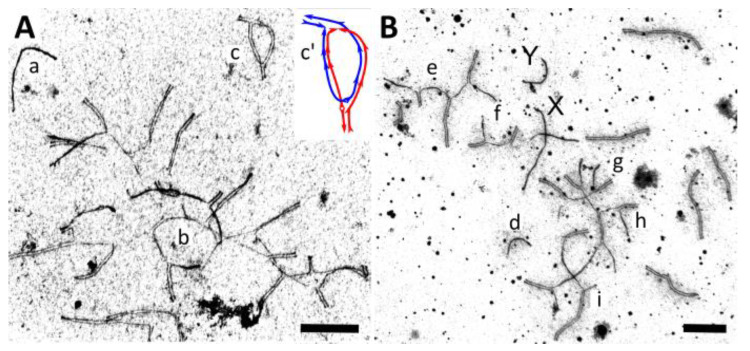
Electron microphotographs of pachytene spermatocytes of interspecies hybrids *A. mujanensis* × *A. maximowiczii*, MUJ × MAX (**A**) and *A. maximowiczii* × *A. evoronensis* “Argi”, MAX × EVA (**B**) stained with silver nitrate. Lowercase letters mark heteromorphic synaptic configurations: univalents: a, d; multivalents: b, e–h; inversion loop and its schematic representation: c, c′. Bar: 5 µm.

The hybrid male MUJ × MAX showed less conspicuous meiotic abnormalities than the other male hybrids (Figure 4G–I; Table 1 and Table S2). Autosomal univalents were rare. The number of different types of synaptic configurations in the hybrid was close to that expected from a comparison of the standard karyotypes of the parental species described by Lemskaya et al. [19,23]. We observed 8–9 homomorphic bivalents, 3 heteromorphic bivalents with misaligned centromeres, 1 heteromorphic bivalent with a large reverse inversion loop (Figure 5A_c_), 1–2 trivalents, and 1–2 complex multivalents involving from 4 to 17 chromosome elements.

The heteromorphic bivalent with a large reverse inversion loop had MLH1 loci inside the loop (Figure 4G). This synaptic configuration could be interpreted as the result of homologous synapsis of two acrocentric chromosomes originating from alternative centromere–telomere fusion of the same ancestral acrocentric chromosomes followed by inactivation of the metacentric centromeres or two large nonoverlapping paracentric inversions in the parental species (Figure 5A).

The multivalent configurations always appeared as chains, although the number of elements involved varied between the cells (Figure 4G–I; Table S5). We detected MLH1 foci at each synapsed SC element (Figure 4G). The number of MLH1 foci was not reduced in the MUJ × MAX hybrids compared to *A. mujanensis* (Table S2; Welch *t*-test, *p* = 0.073). A small number of unsynapsed SC regions lacking SYCP1 (Figure 4F) was labeled with γH2A.X antibodies (Figure 4I). We suggest that almost complete synapsis and recombination enabled a fraction of the spermatocytes to undergo further spermatogenesis progression. However, as our histological analysis shows, these cells were arrested at the end of spermatogenesis, apparently due to the transcriptional inactivation of unsynapsed chromatin at earlier stages of spermatogenesis.

The male hybrids of *A. evoronensis* with *A. mujanensis* and *A. maximowiczii* showed more pronounced asynapsis and recombination suppression (Figure 4J–O). Two reciprocal hybrids between MUJ and EVA, three EVA × MAX hybrids, and three MAX × EVE hybrids examined showed almost no sign of MLH1 signals (Figure 4J,M). In one EVA × MAX male, some germ cells reached a mid-pachytene-like stage and displayed MLH1 foci at almost all synapsed arms (Tables S2 and S6), therefore the mean number of MLH1 signals per cell in this hybrid did not differ from that of the parental species (Welch *t*-test, *p* < 0.1). We detected no differences in synaptic progression between the reciprocal hybrids EVA × MUJ and MUJ × EVA. There were about 8–9 homomorphic bivalents, 1–2 heteromorphic bivalents, and 1–4 complex multivalents involving from 5 to 21 chromosome elements in the average spermatocyte. Cells differ in the number of multivalent chains and the number of SC elements involved in each of them (Figure 4J–L) (Tables S2 and S5). Most of the chains showed extensive asynapsis in their terminal arms and the pericentromeric regions of the internal arms. We also observed unpaired univalents (Figure 5B). They, as well as the multivalents involving a small number of elements, apparently occurred due to topological difficulties in assembling complete chains involving many chromosomes with partial homology.

The univalents and asynapsed regions of the multivalents showed no SYCP1 signal (Figure 4J) and were heavily labeled with γH2A.X clouds (Figure 4L), indicating the widespread occurrence of unrepaired DSBs and meiosis-specific inactivation of unsynapsed chromatin. Apparently, these aberrations triggered the apoptosis of the primary spermatocytes, which we observed during the histological examination of these specimens.

The most severe abnormalities were observed in one EVA × MAX male hybrid. Meiosis did not advance beyond the zygotene-like stage. In this hybrid, most bivalents and all multivalents displayed extended asynapsis. There were at least 11 autosomal univalents per cell. In some cells, all chromosomes were asynapsed (Figure 4M–O).

Thus, chromosome synapsis was disrupted in all of the interspecies hybrids (Figure 4 and Figure 5; Table 1). The most common aberration was complete or partial asynapsis of the homologous chromosomes in the bivalents and the complex multivalent chains. It led to a delayed repair of DNA double-strand breaks and the silencing of unpaired chromatin marked by γH2A.X antibodies. However, in most of the synapsed chromosome regions, we detected MLH1 foci marking homologous recombination. This indicates rather high degree of homology between the DNA sequences of the homeologous chromosome regions of the recently diverged species of the genus *Alexandromys*.

## 4. Discussion

### 4.1. Interspecies Hybrids Are Sterile, Whereas Intraspecies Hybrids Show Normal Reproductive Potential

The histological analysis shows that the male hybrids between closely related but karyotypically divergent species of *A. maximowiczii*, *A. mujanensis,* and *A. evoronensis* are unable to produce viable gametes. These findings align with those of Meyer et al. [21], who also investigated these hybrids and found complete sterility and severe disruption of gametogenesis, similar to our results. Our study suggests that male-hybrid sterility is caused by the arrest of spermatogenesis primarily at the stage of early primary spermatocyte, followed by apoptosis of the germ cells, as well as multiple abnormalities of the few surviving spermatids and spermatozoa. In contrast, we observed normal spermatogenesis in chromosomally heterozygous males of *A. maximowiczii* and *A. evoronensis*, as well as in hybrids between the geographically isolated populations and chromosome races of *A. evoronensis*, indicating their potential fertility. What is the cytological basis for these differences in fertility between inter- and intraspecies hybrids?

### 4.2. Asynapsis Is the Main Cause of Meiotic Arrest and Sterility in Interspecies Hybrids

The results of our cytogenetic analysis indicate that the main cause of meiotic arrest and sterility in the interspecies hybrids of *A. maximowiczii*, *A. evoronensis*, and *A. mujanensis* is asynapsis of homeologous chromosomes, visualized by immunolocalization of the proteins of lateral and central elements of SC (SYCP3 and SYCP1) (Figure 4) and by electron microscopy of silver-stained SC (Figure 5).

It has been shown in several species of mammals that asynapsis leads to the accumulation of unrepaired recombination intermediates and, what is more, unpaired chromosome regions undergo transcriptional silencing [34,45]. The silencing of genes that are necessary for the meiotic progression of pachytene spermatocytes may lead to immediate or deferred meiotic arrest [34,46]. This, in turn, triggers apoptosis [47,48].

In the interspecies hybrids of *A. maximowiczii*, *A. evoronensis*, and *A. mujanensis*, we observed all these consequences of extensive asynapsis: accumulation of unrepaired recombination intermediates and meiotic silencing of unsynapsed chromatin marked by γH2A.X antibodies (Figure 4), apoptosis of primary spermatocytes detected by TUNEL (Figure 1), and various postmeiotic aberrations such as a reduced number, and abnormal morphology, of spermatids and spermatozoa (Table 1).

### 4.3. Asynapsis in Interspecies Hybrids Is Due to Complex Heterozygosity for a Series of Chromosome Rearrangements

All interspecies hybrids examined here were complex heterozygotes for a series of alternative chromosome fusions with monobrachial homology. Different fusions of different acrocentric chromosomes in different combinations (Robertsonian or tandem in different orders and orientations) have been fixed or remain polymorphic in the parental species [23,24,28]. Homologous synapsis of these chromosomes in the hybrids would result in the formation of long (up to 21-element) chains. Topological constraints prevent synapsis in the pericentromeric regions of the chains and lead to the fragmentation of the expected long chain of chromosomes with monobrachial homology into several shorter chains with asynaptic ends (Figure 4 and Figure 5). Complex heterozygosity for pericentric inversions and centromeric shifts in the arms involved in these chains amplified the constraints. The variation in the complexity of synaptic configuration determined the variation in the severity of meiotic aberrations between the types of interspecies hybrids and between the hybrid offspring of the same crosses.

Asynapsis is the main, but not the only cause, of the sterility in vole hybrids. The complexity of the synaptic configurations might lead to chromosome nondisjunction in the small number of germ cells advanced until MI and the production of unbalanced gametes. Meyer et al. [21] reported complex multivalent chains at MI in the MUJ × MAX male hybrids.

Our finding is in line with the results of studies on the effects of simple and complex heterozygosity for a series of Robertsonian translocations on meiotic chromosome behavior and fertility in wild and laboratory mice [12,49,50,51,52,53,54,55,56,57,58].

### 4.4. Simple Heterozygosity for Several Chromosome Rearrangements Does Not Disrupt Chromosome Synapsis and Recombination in the Intraspecies Hybrids of A. evoronensis

Interpopulation and interracial hybrids of *A. evoronensis* demonstrated orderly chromosome synapsis and normal fertility (Figure 5). All of them were simple heterozygotes for three or more (up to nine) chromosome rearrangements. Asynapsis in these hybrids was rare, restricted by small chromosome regions, and transient. Before the end of pachytene, it was usually replaced by nonhomologous synapsis (Figure 3 and Figure 4; Figures S1 and S2).

Simple heterozygotes for several chromosome fusions of *A. evoronensis* interracial hybrids sometimes showed delayed synapsis in pericentromeric regions. Apparently, it has been caused by topological difficulties in the presynaptic alignment of the chromosomes involved. However, the asynapsis was transient and usually disappeared before the end of pachytene. The pericentromeric regions of the twin acrocentrics were either homologously synapsed with the metacentric partner or nonhomologously synapsed with each other, forming a side arm. In the latter case, the metacentric partner was shortened via synaptic adjustment. Similarly, little or no effect of single and multiple heterozygosity for chromosome fusions on chromosome synapsis has been demonstrated in such chromosomally polymorphic species, such as the tuco-tuco [59,60] and the common shrew [8,61].

Thus, transient asynapsis in the pericentromeric regions of the trivalents in simple heterozygotes for chromosome fusions does not affect the fertility of the carriers.

The modern version of the chromosome speciation hypothesis emphasizes the importance of recombination suppression around the breakpoints of the rearrangements for the restriction of gene flow via hybrids. This factor is unlikely to contribute to the speciation of gray voles. The recombination rate is maintained at a minimal level of one chiasma per chromosome on average in the species examined here and the other species of the genus *Microtus* described earlier [62,63,64]. Moreover, chiasmata are located close to the distal ends of the vole chromosomes. In this study, we did not detect a substantial decrease in recombination in the interracial hybrids of *A. evoronensis* and interspecies MUJ × MAX hybrids. Asynapsis and meiotic arrest in other interspecies vole hybrids led to almost complete suppression of recombination (Table 1). However, it did not matter, since hybrid sterility suppressed gene flow via the hybrids in a more efficient way than recombination suppression.

### 4.5. Chromosome Polymorphism of the “Maximowiczii” Group of Species Is Apparently Neutral

The results of our study show that simple heterozygosity for several chromosome rearrangements does not disrupt chromosome synapsis and meiotic progression in the carriers. This indicates that selection against chromosomal heterozygotes is unlikely. The recombination suppression in simple heterozygotes for inversions, centromere shifts, and chromosome fusions might enforce linkage disequilibrium between the alleles located in the affected regions. Such pericentromeric blocks of tightly linked alleles (supergenes) have been found in several species of plants and animals [65]. However, as we mentioned above, the recombination rate in these species is already rather low and biased toward the chromosome ends. Therefore, we doubt that the subtle changes in recombination patterns of the simple heterozygotes might be responsible for the stable maintenance of the chromosome polymorphism in the species examined. This suggestion would demand an unrealistically strong selective advantage of the chromosomal heterozygotes.

Population subdivision inside the species examined is a more plausible explanation of the chromosome polymorphisms in each of them. The known ranges of *A. evoronensis* and *A. mujanensis* are patchy and probably grossly underestimated. *A. maximowiczii* shows a relatively wide but mosaic distribution [17]. Yet, each of the three local populations of *A. evoronensis* studied so far is chromosomally polymorphic and differs from the others both karyotypically and genetically [27]. Three local populations of *A. mujanensis* differ in inversions [23,24]. *A. maximowiczii* includes at least twenty different karyotypes [18].

One might suggest that each of the species is a mosaic of small, partially overlapping, but karyotypically different populations. Such a population structure is characteristic of several genera of rodents: *Ctenomys*, *Ellobius*, *Spalax*, etc. [66,67,68]. In this case, chromosomal heterozygotes observed in species of the “*maximowiczii*” group can be interpreted as the results of recent hybridization between neighboring local populations.

Sporadic contacts between nonhomologous chromosomes during meiotic prophase in the interpopulation and interracial hybrids heterozygous for several chromosome fusions might induce the occurrence of new chromosome rearrangements, as suggested by Matveevsky et al. [69]. This, in turn, might promote karyotypic divergence between the species.

### 4.6. Karyotypic Divergence Promotes Speciation in the “Maximowiczii” Group of Species

The molecular clock analysis suggests that the group of species including *A. maximowiczii*, *A. evoronensis*, and *A. mujanensis* diverged from the rest of the *A. maximowiczii* species complex about 110 thousand years ago [29,30]. Yet, they accumulated rather substantial karyotypic differences and reached complete intrinsic reproductive isolation from each other [21]. The results of our cytological analysis suggest that karyotypic divergence between the parental species is the most probable cause of meiotic arrest and sterility in their male hybrids.

It is interesting to compare meiotic chromosome behavior in the sterile hybrids between the “maximowiczii” group of species examined here and between the “arvalis” group of species examined earlier by Torgasheva and Borodin [64]. The estimated time since the last common ancestor of two species of the “arvalis” group (*Microtus arvalis* and *M. rossiaemeridionalis*) is twice as long: about 200 thousand years. Their karyotypes differ by four tandem fusions, one paracentric, and six pericentric inversions, and four to seven putative centric transpositions. Therefore, their hybrids are simple heterozygotes for 15–18 rearrangements. They are expected to form four trivalents and 11–14 heteromorphic bivalents.

Torgasheva and Borodin [64] found that in male *M. arvalis* × *M. rossiaemeridionalis* hybrids, meiosis was arrested at the leptotene, while in female hybrids, it reached the pachytene. The majority of the hybrid oocytes contained many univalents and multivalents involving nonhomologous chromosomes with extended regions of asynapsis, while some of the oocytes showed almost perfect chromosomal pairing. The wide variation between genetically and chromosomally identical oocytes in the ratio of homologously paired to nonhomologously paired regions was interpreted as evidence that the choice between homologous and nonhomologous synapsis was random. Torgasheva and Borodin [64] suggested that significant differences in DNA sequences between the parental species impeded the homology search and the timely eradication of ectopic interactions between the nonhomologous chromosomes in the meiotic prophase of the hybrids.

A somewhat similar situation has been described in the hybrids between several other species of the *M. arvalis* group [63]. In the hybrids between *M. kermanensis* and *M. transcaspicus* which differ in eight chromosomal rearrangements [70], meiosis stops at the leptotene stage. The synapsis in these male hybrids does not start at all, while in hybrids between *M. arvalis* and *M. kermanensis*, which differ in 14 chromosomal rearrangements [70], meiosis stops at the zygotene stage, showing a series of synaptic disruptions.

Thus, genetic divergence has been considered the main cause of the synaptic disturbances in the hybrids between the “arvalis” group of species. Chromosomal divergence has been treated as a subsidiary factor, that might affect presynaptic alignments of the homologs and aggravate the difficulties in homology search [63,64].

On the contrary, in the species of the “maximowiczii” group, extreme chromosomal divergence plays the main role in disrupting homologous synapsis in their interspecies hybrids. Univalents are rare. Most asynapsis occurs in the pericentromeric regions and in the terminal arms of the incomplete chains with monobrachial homology. Genetic homology between the DNA sequences of different species remains rather high, as evidenced by the presence of recombination nodules in all homomorphic and heteromorphic bivalents, and most synapsed segments in the multivalents. Thus, we may suggest that the accumulation of multiple chromosome rearrangements in small, isolated populations played an important role in speciation in the “maximowiczii” group of species.

## Figures and Tables

**Table 1:** Abnormalities of gametogenesis in Eastern Asian voles *Alexandromys* and their hybrids.

Dam	Sire	N	Most Advanced Stage of Spermatogenesis	Spermatogenesis	Meiotic Prophase	Mean Number of MLH1 Foci per Cell
**Parental crosses**						
MAX	MAX	4	Spermatozoa	Normal	Normal	23.9 ± 2.2
MUJ	MUJ	3	Spermatozoa	Normal	Normal	20.2 ± 1.6
EVA	EVA	4	Spermatozoa	Normal	Normal	20.7 ± 2.0
EVE	EVE	2	Spermatozoa	Normal	Normal	22.0 ± 1.1
**Interpopulation and interracial crosses**						
EVA2	EVA1	2	Spermatozoa	Normal	Normal	20.5 ± 1.5
EVA	EVE	3	Spermatozoa in two males, early spermatocytes I in one male	Normal in two males, delayed spermatogenesis in one male	Normal	21.1 ± 1.3
**Interspecies crosses**						
MUJ	MAX	1	Spermatozoa	Spermatid balls, abnormal spermatozoa	Univalents, heteromorphic, and partially asynaptic bivalents, complex multivalents, γH2A.X at asynapsed regions, MLH1 foci at each synapsed element	21.3 ± 3.3
EVA	MUJ	2	Spermatozoa	Spermatid balls and abnormal spermatozoa in one male, sporadic normal spermatids in another male	Univalents, heteromorphic, and partially asynaptic bivalents, complex multivalents, γH2A.X at the asynapsed regions, few MLH1 foci.	-
MUJ	EVA	1	Spermatozoa	Arrest at a pachytene-like stage	Univalents, heteromorphic, and partially asynaptic bivalents, complex multivalents, γH2A.X at the asynapsed regions, no MLH1 foci.	-
MAX	EVE	3	Early spermatocytes I	Arrest at a pachytene-like stage	Univalents, heteromorphic, and partially asynaptic bivalents, complex multivalents, γH2A.X at the asynapsed regions, no MLH1 foci.	-
EVA	MAX	4	Early spermatocytes I	Arrest at zygotene stage mostly	Univalents, heteromorphic, and partially asynaptic bivalents, multivalents with extended asynapsis, γH2A.X at the asynapsed regions, MLH1 foci detected in 4 cells.	23.3 ± 2.2

## Data Availability

All data generated or analyzed during this study are included in this published article and its Supplementary Information files.

## Supplementary Materials

The following supporting information can be downloaded at: https://www.mdpi.com/article/10.3390/genes14051022/s1, Figure S1: Synaptic adjustment of heteromorphic bivalent in *A. maximowiczii* (MAX) spermatocytes revealed by immunolocalization of SYCP3 (red), MLH1 (green), and centromeric proteins (blue); Figure S2: Pachytene spermatocytes of interpopulation *A. evoronensis* “Argi”2 × *A. evoronensis* “Argi”1 (EVA2 × EVA1) hybrid (**A**) and interracial *A. evoronensis* “Argi”2 × *A. evoronensis* “Evoron” (EVA2 × EVE) hybrid (**B**) after immunolocalization of SYCP3 (red), MLH1 (green), and centromeric proteins (blue). Lowercase letters mark the heteromorphic bivalents and trivalents, as described in the text. Arrowheads indicate the centromeres of the heteromorphic bivalents and trivalents. Bar: 10 µm; Table S1: List of the species examined; Table S2: Summary of cytogenetic characteristics of the specimens and genetic groups; Table S3: Information about the frequency of spermatocytes (SPC) and spermatids (SPTD) in the testes of F1 intra- and interspecies vole hybrids and *A. evoronensis*; Table S4: Information about the apoptotic index of spermatocytes (SPC) and spermatogonia (SPG) in the testes of F1 intra- and interspecies vole hybrids and *A. mujanensis*; Table S5: Synaptic characteristics of the specimens; Table S6: MLH1 focus number per cell.
