# Supplementary figures and images for: Chromosome Asynapsis Is the Main Cause of Male Sterility in the Interspecies Hybrids of East Asian Voles (Alexandromys, Rodentia, Arvicolinae)
Source: Genes (Basel). 2023 Apr 30;14(5):1022. doi: 10.3390/genes14051022 (PMC10218298; doi:10.3390/genes14051022)

**SYCP3**  
**MLH1**  
**ACA**

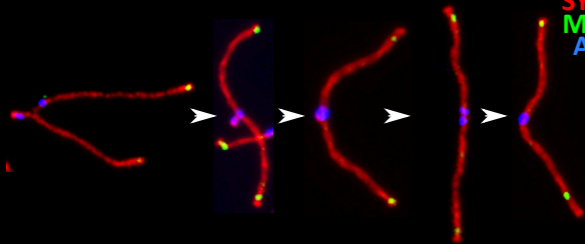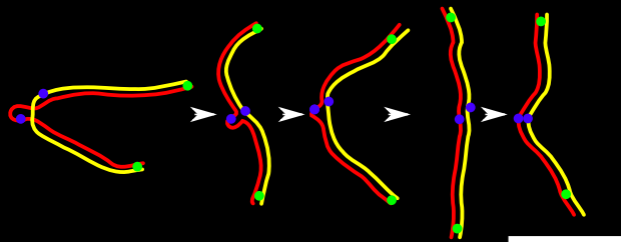

Supplement: Supplementary file 1 [file genes-14-01022-s001.zip › Hybrid_sterility_Supplementary_materials/Figure S1.pdf]

**A**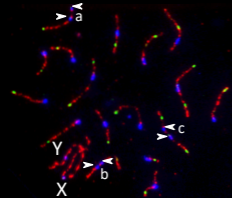**B**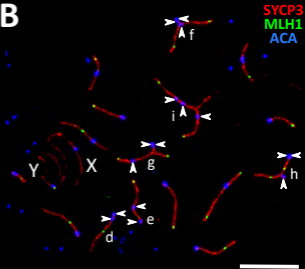

Supplement: Supplementary file 1 [file genes-14-01022-s001.zip › Hybrid_sterility_Supplementary_materials/Figure S2.pdf]
